# The influence of neighbourhood-level socioeconomic deprivation on cardiovascular disease mortality in older age: longitudinal multilevel analyses from a cohort of older British men

**DOI:** 10.1136/jech-2015-205542

**Published:** 2015-08-18

**Authors:** S E Ramsay, R W Morris, P H Whincup, S V Subramanian, A O Papacosta, Lucy T Lennon, S G Wannamethee

**Affiliations:** 1 Department of Primary Care & Population Health, UCL, London, UK; 2 Division of Population Health Sciences and Education, St George's University of London, London, UK; 3 Department of Social and Behavioural Science, Harvard University, Boston, Massachusetts, USA

**Keywords:** DEPRIVATION, AGEING, Cardiovascular disease, INEQUALITIES, MULTILEVEL MODELLING

## Abstract

**Background:**

Evidence from longitudinal studies on the influence of neighbourhood socioeconomic factors in older age on cardiovascular disease (CVD) mortality is limited. We aimed to investigate the prospective association of neighbourhood-level deprivation in later life with CVD mortality, and assess the underlying role of established cardiovascular risk factors.

**Methods:**

A socially representative cohort of 3924 men, aged 60–79 years in 1998–2000, from 24 British towns, was followed up until 2012 for CVD mortality. Quintiles of the national Index of Multiple Deprivation (IMD), a composite score of neighbourhood-level factors (including income, employment, education, housing and living environment) were used. Multilevel logistic regression with discrete-time models (stratifying follow-up time into months) were used.

**Results:**

Over 12 years, 1545 deaths occurred, including 580 from CVD. The risk of CVD mortality showed a graded increase from IMD quintile 1 (least deprived) to 5 (most deprived). Compared to quintile 1, the age-adjusted odds of CVD mortality in quintile 5 were 1.71 (95% CI 1.32 to 2.21), and 1.62 (95% CI 1.23 to 2.13) on further adjustment for individual social class, which was attenuated slightly to 1.44 (95% CI 1.09 to 1.89), but remained statistically significant after adjustment for smoking, body mass index, physical activity and use of alcohol. Further adjustment for blood pressure, high-density lipoprotein cholesterol and prevalent diabetes made little difference.

**Conclusions:**

Neighbourhood-level deprivation was associated with an increased risk of CVD mortality in older people independent of individual-level social class and cardiovascular risk factors. The role of other specific neighbourhood-level factors merits further research.

## Introduction

Cardiovascular disease (CVD) remains the main cause of death in the UK, particularly at older ages.^1^ Therefore, with the increasing proportion of older people in countries such as those in the UK, CVD poses a significant public health challenge.^1^ Apart from the overall mortality burden, social inequalities also persist, with higher CVD mortality rates among people from lower compared with higher socioeconomic groups.^1^ ^2^ Studies have also shown that neighbourhood-level socioeconomic factors are associated with CVD mortality—people living in more deprived or disadvantaged areas are reported to have a greater risk of CVD mortality.^3–9^ However, most studies on neighbourhood-level socioeconomic factors and CVD mortality have been in middle-aged populations (table 1 for a summary of studies).

**Table 1:** Studies investigating associations between neighbourhood-level socioeconomic factors and cardiovascular disease mortality

Reference	Age of participants, years	Setting	Area or neighbourhood socioeconomic measure used	Relative risks (95% CI)
Smith *et al*^4^	45–64	Renfrew and Paisley, Scotland	Carstairs deprivation score (based on male unemployment, overcrowding, car ownership, proportion in social classes IV and V)	HR for most deprived vs least deprived categories 1.26 (1.04 to 1.52) for men 1.33 (1.05 to 1.69) for women
Waitzman and Smith^9^	25–74	USA—National Health and Nutrition Examination Survey	Federally defined poverty areas of residence based on census tracts	RR for poverty-area vs non-poverty area 1.90 (1.24 to 2.90) in 25–54 years 0.83 (0.66 to 1.03) in 55–74 years
Diez Roux *et al*^5^	≥65	USA—Forsyth Co, North Carolina; Washington Co, Maryland; Sacramento Co, California and Pittsburgh, Pennsylvania	Neighbourhood deprivation score based on census (household income, value of housing units, education and occupation)	HR for most vs least disadvantaged tertiles 1.5 (1.2 to 1.9) in Caucasian participants 1.2 (0.7 to 2.2) in African–American participants
Borrell *et al*^3^	45–64	USA—Forsyth County, North Carolina; Jackson, Mississippi; the northwestern suburbs of Minneapolis, Minnesota; and Washington County, Maryland	Neighbourhood deprivation score based on census (household income, value of housing units, education and occupation)	HR for most vs least disadvantaged tertiles 1.4 (1.0 to 2.0) in Caucasian participants 1.1 (0.8 to 1.6) in African-American participants
Steenland *et al*^8^	50–74	USA—Cancer Prevention Study II Nutrition Cohort	Area-level socioeconomic status based on census data including household income, home value, occupation and education	RR for lowest vs highest area-level score group 1.46 (1.22 to 1.74) for men 1.33 (1.00 to 1.77) for women
Major *et al*^6^	50–71	USA—California, Florida, Louisiana, New Jersey, North Carolina, Pennsylvania, Atlanta (Georgia) and Detroit (Michigan)	Neighbourhood deprivation index based on census data (housing, residential stability, poverty, employment, occupation, racial composition, education)	HR for highest vs lowest deprivation quintile 1.33 (1.19 to 1.49) for men 1.18 (1.01 to 1.38) for women
Sanchez-Santos *et al*^7^	60–79	24 British towns	Index of multiple deprivation (income, employment, barriers to services, living environment)	HR per SD increase in deprivation score 1.22 (1.09 to 1.37) in women
Chan *et al* 2014^12^	All ages	USA—458 counties	Community characteristics including US census data	Estimated increase in death per 100 000 from 25th to 75th centile—for education 19.92 (14.12 to 25.80); 16.06 (10.77 to 21.45) for employment in construction

HR, hazard ratio; RR, rate ratio.

There is an increasing interest in understanding the influence of neighbourhood-level socioeconomic factors particularly on the health of older populations^10^—it has been suggested that socioeconomic factors of neighbourhoods (such as housing, living environment, access to services) are likely to play an important role in older people who are more confined to their area of residence.^10^ We have previously shown that individual-level socioeconomic position is associated with CVD even in older age.^11^ However, there is limited evidence from longitudinal studies of older people assessing the influence of neighbourhood socioeconomic factors on CVD mortality.^5^ ^7^ ^9^ We therefore aimed to investigate the independent relationship between neighbourhood (or area)-level socioeconomic factors and CVD mortality, and all-cause mortality, over a 12 year period in a representative sample of older British men. We also explored the possible role of individual socioeconomic position and established behavioural cardiovascular risk factors (including smoking, obesity, physical activity) in explaining associations between neighbourhood socioeconomic factors and CVD mortality.

## Methods

The British Regional Heart Study (BRHS) is a longitudinal study comprising a socially and geographically representative sample of 7735 men recruited from general practices in 24 British towns. Since the sample frame excluded inner cities and highly mobile towns, the study population comprised almost completely of white European participants. The cohort has been followed up since 1978–1980, when aged 40–59 years. All men provided written informed consent to the investigations, carried out in accordance with the Declaration of Helsinki. Ethical approval was provided by relevant local research ethics committees throughout. Between 1998 and 2000, the men, aged 60–79 years, were invited for a follow-up examination. This reassessment included completion of a questionnaire on lifestyle and medical history, and a physical examination including measurements of anthropometry and blood pressure (BP). Blood samples were collected after a minimum 6 h fast, using the Sarstedt Monovette system. Of the surviving participants 4252 men attended the examination, a response rate of 77%, with a slightly higher response rate of 80% in non-manual social classes and 70% in manual groups; 4045 men had biochemical measurements. Details of cardiovascular risk factors in the cohort assessed at this examination at 60–79 years (smoking, physical activity, body mass index (BMI), alcohol intake, BP, blood lipids and glucose) have been described.^13^ ^14^ Details of the use of these risk factors in this study are reported in the statistical analyses section below.

### Neighbourhood-level socioeconomic deprivation

The Indices of Multiple Deprivation (IMD) for England (2004),^15^ Scotland (2004)^16^ and Wales (2005),^17^ were used as measures of neighbourhood-level socioeconomic deprivation. These national scores of deprivation are collected at aggregate level for small geographical units called ‘super output areas’, of which the lower super output area (LSOA) is the smallest with an average of 1500 people; small area units in Scotland are called ‘Data Zones’ (average of 750 people). The overall IMD is conceptualised as a weighted area-level aggregation of different ‘domains’ or aspects of deprivation, with a higher score indicating greater deprivation. Each IMD domain is based on specific area-level indicators mostly from 2000 to 2001, and therefore applicable to this cohort data from 1998 to 2000. The English IMD 2004 includes income, employment, health and disability, education, skills and training, barriers to housing and services, living environment and crime. The Scottish IMD 2004 comprises similar domains but includes ‘geographic access and telecommunications’ instead of ‘barriers to housing’, ‘living environment’ and ‘crime’. The Welsh IMD 2005 comprises income, employment, education, health, access to services, housing and environment. IMD scores for the BRHS cohort were based on LSOAs derived from postcodes of residence at 60–79 years.^18^ Since the BRHS cohort comprises men from England, Scotland and Wales, the IMD scores were standardised to obtain a combined IMD measure.^19^

### Individual-level socioeconomic position

The longest-held occupation of participants at study entry (aged 40–59 years) was used to define social class using the Registrar Generals’ Social Class Classification—I (professionals, eg, physicians, engineers), II (managerial, eg, teachers, sales managers), III non-manual (semiskilled non-manual, eg, clerks, shop assistants), III manual (semiskilled manual, eg, bricklayers), IV (partly skilled, eg, postmen) and V (unskilled, eg, porters, general labourers).

### Follow-up

The cohort has been followed up for mortality through the National Health Service Central Register. CVD deaths included those with International Classification of Diseases, ninth revision (ICD-9) codes of 401–459. Follow-up for this study was for 12 years until 2012. Non-fatal myocardial infarction (MI) and non-fatal stroke were identified from biennial reviews of the general practitioner records of study participants, which include hospital and clinic correspondence, and information on diagnoses of diseases including MI and stroke. Major CVD incidence included non-fatal MI, non-fatal stroke and CVD deaths.

### Statistical analyses

An adjusted IMD score was applied to the cohort, using the employment and income IMD domains, which are common to the English, Scottish and Welsh IMDs, and measured with similar indicators.^19^ We obtained component IMD scores for each country including the income and employment scores expressed as percentages. For each country, a linear regression of the overall IMD score on employment and income was carried out. The coefficients for employment and income, and residuals after fitting to the English overall IMD, were applied to that of the Scottish and Welsh component scores, to obtain adjusted IMD scores for Scotland and Wales. These adjusted IMD scores for England, Scotland and Wales combined were divided into quintiles from the least to most deprived quintile, and were applied to the BRHS cohort.

Multilevel modelling was used to take account of hierarchies in the data, with individuals nested within LSOAs. IMD quintiles were level 2 variables, while individual social class and risk factors were level 1 variables. The cohort comprised 1674 LSOAs with an average of two men in each LSOA (941 LSOAs had 1 participant; 297 had 2; 354 had 3–6 and 82 had 7–16 participants). Multilevel discrete-time models were used^20–22^ for the longitudinal data, whereby the 12 year follow-up was divided into 1-month discrete intervals. Multilevel logistic regression analyses were carried out for mortality (all-cause and CVD) and included discrete-time intervals in the regression model by fitting suitable polynomial functions of time to obtain a smoothed hazard function.^20–22^ ORs (which approximated HRs within follow-up months) and 95% CIs were obtained according to quintiles of IMD deprivation, with quintile 1 (least deprived) as the baseline group. Of the 4045 participants with biological measures, those of armed forces occupation or without information on social class (n=114) were excluded from the analyses; a small number of men living overseas (n=7) who were not allocated a LSOA were also excluded from the analyses. Therefore, analyses were carried out on 3924 participants. Intraclass correlation coefficients (ICC) were calculated to obtain the variance explained in mortality by the area-level variable (LSOAs).^23^ Subsidiary analyses were carried out for CVD incidence and for non-CVD mortality as outcomes. The models were adjusted for age followed by further sequential adjustments for individual social class: smoking, alcohol consumption, physical activity and BMI; and, finally, systolic BP, high-density lipoprotein cholesterol (HDL-C) and diabetes. For the adjustments, age, systolic BP, BMI and HDL-C were fitted as continuous variables. Social class (6 levels of social classes I, II, III non-manual, III manual, IV, V), smoking (seven levels of never, long-term ex-smoker >20 years, long-term ex-smoker for 15–20 years, ex-smoker for 10–15 years, ex-smoker for 5–10 years, gave up in last 5 years and current smoker), physical activity (six levels of inactive, occasional, light, moderate, moderate-vigorous, vigorous), alcohol intake (five levels of none, occasional, light, moderate, heavy) and marital status (married and unmarried including single, divorced or widowed) were fitted as categorical variables in adjusted models. Age-adjusted models were also carried out according to quintiles of the separate components of the English IMD (this analysis was restricted to English IMD due to differences in the components of English, Scottish and Welsh IMDs). Sensitivity analyses restricted to participants from England (n=3374) were carried out to compare results from the main analyses using the combined adjusted IMD quintiles with results for English IMD quintiles. All analyses were carried out using SAS V.9.3, MLwiN 2.29^24^ and Stata V.13 (using the ‘runmlwin’ command).^25^

## Results

Among 3924 men aged 60–79 years, 1545 deaths occurred during the 12 year follow-up period, of which 580 were from CVD. Table 2 shows the distribution of age, individual social class and established cardiovascular risk factors according to IMD quintiles. The proportion of men of manual social class and those with ≥3 adverse socioeconomic indicators increased from quintile 1 (least deprived) to quintile 5 (most deprived). Proportions of current smokers, physically inactive, moderate/heavy drinkers and obese men were also higher in the more deprived quintiles, while HDL-C levels were lower. Systolic BP or diabetes did not vary by IMD quintiles.

**Table 2:** Baseline characteristics according to neighbourhood-level deprivation quintiles in a cohort of British men aged 60–79 years

	Neighbourhood-level deprivation (Index of Multiple Deprivation)					p Value for trend
	Quintile 1 (least deprived) (n=900)	Quintile 2 (n=918)	Quintile 3 (n=774)	Quintile 4 (n=670)	Quintile 5 (most deprived) (n=662)	
Mean, age (SD)	68 (5)	68 (5)	68 (6)	69 (6)	69 (5)	0.006
Manual occupational social class, n (%)	262 (29)	382 (42)	434 (56)	467 (70)	520 (79)	<0.0001
≥3 Adverse socioeconomic factors,* n (%)	45 (5)	87 (9)	154 (20)	200 (30)	267 (40)	<0.0001
Current smokers, n (%)	69 (8)	85 (9)	93 (12)	108 (16)	136 (21)	<0.0001
Moderate/heavy drinkers,† n (%)	151 (17)	174 (19)	134 (17)	122 (18)	154 (23)	0.01
Physically inactive, n (%)	250 (28)	272 (30)	262 (34)	255 (38)	258 (40)	<0.0001
Obese (BMI ≥30 kg/m^2^), n (%)	111 (12)	148 (16)	125 (16)	136 (20)	134 (20)	<0.0001
Mean systolic blood pressure, mm Hg (SD)	149 (24)	149 (25)	149 (24)	150 (24)	149 (24)	0.89
Prevalent diabetes—n (%)	93 (10)	91 (10)	92 (12)	88 (13)	85 (13)	0.16
Mean HDL cholesterol, mmol/l (SD)	1.36 (0.34)	1.34 (0.35)	1.31 (0.34)	1.29 (0.32)	1.30 (0.35)	<0.0001

*Score includes: no car, not house owner, state pension only, no central heating, manual SC, education ≤14 years.

†>16 drinks of alcohol per week (1 UK unit=10 g).

BMI, body mass index; HDL, high-density lipoprotein.

OR (95% CI) for CVD mortality according to neighbourhood-level deprivation quintiles are presented in table 3. Compared to quintile 1, the risk of CVD mortality showed a graded increase in more deprived quintiles. The estimates weakened slightly but remained statistically significant on adjustment for individual social class. A formal test of interaction between IMD deprivation and social class showed no evidence that the effect of deprivation varied by social class (p for test for interaction=0.66). The increased risk in more deprived quintiles was attenuated slightly after additional adjustment for behavioural risk factors (smoking, physical activity and BMI), and remained unchanged on further adjustment for systolic BP, HDL-C and diabetes. The increased risks of CVD mortality in quintiles 3, 4 and 5 remained significant in the final fully adjusted model. Further adjustment for marital status made no material difference to these estimates (data not shown). No difference in risk of CVD incidence (including non-fatal and fatal cases) according to IMD quintiles was observed—adjusted for age and individual social class, compared to quintile 1; OR (95% CI) for CVD incidence were 1.09 (0.88 to 1.34) for quintile 2; 1.25 (1.01 to 1.55) for quintile 3; 1.20 (0.95 to 1.50) for quintile 4 and 1.16 (0.92 to 1.47) for quintile 5.

**Table 3:** ORs (95% CI) for cardiovascular disease mortality according to neighbourhood-level deprivation quintiles in a cohort of British men aged 60–79 years followed up for 12 years

	Number of deaths (%)	Adjusted for age	Further adjusted for individual social class	Further adjusted for smoking, physical activity, alcohol, body mass index	Further adjusted for high-density lipoprotein cholesterol, systolic blood pressure and diabetes
Quintile 1 (n=900)	99 (11)	1.00	1.00	1.00	1.00
Quintile 2 (n=918)	118 (13)	1.21 (0.93 to 1.56)	1.17 (0.90 to 1.51)	1.15 (0.89 to 1.50)	1.18 (0.90 to 1.56)
Quintile 3 (n=774)	135 (17)	1.64 (1.28 to 2.11)	1.58 (1.22 to 2.04)	1.50 (1.16 to 1.94)	1.58 (1.20 to 2.07)
Quintile 4 (n=670)	107 (16)	1.57 (1.21 to 2.03)	1.49 (1.14 to 1.96)	1.34 (1.02 to 1.77)	1.38 (1.03 to 1.84)
Quintile 5 (n=662)	121 (18)	1.71 (1.32 to 2.21)	1.62 (1.23 to 2.13)	1.44 (1.09 to 1.89)	1.43 (1.07 to 1.92)
Random effect variance (SE)		0.00	0.0009 (0.06)	0.003 (0.06)	0.00
Intraclass correlation coefficient (ICC)		0	0.0003	0.000003	0
P for trend		<0.0001	<0.0001	0.006	0.01

Results for all-cause mortality according to IMD quintiles of neighbourhood deprivation are presented in table 4. The risk of all-cause mortality showed a graded increase from the least deprived (quintile 1) to the most deprived quintile (quintile 5). These increased risks in more deprived quintiles remained statistically significant, with little change in estimates after adjustment for individual social class and risk factors. A test for interaction showed no evidence that the effect of deprivation varied by social class (p for test for interaction=0.77). The random effect variance for LSOA (measure of neighbourhood) was not statistically significant; the corresponding ICCs were also very small, indicating that the neighbourhoods explained little of the variance in mortality. Associations between neighbourhood IMD quintiles and non-CVD mortality were weaker than those with CVD mortality. A greater risk for non-CVD mortality was observed in quintiles 4 and 5—ORs (95% CI) were 1.37 (1.12 to 1.69) and 1.42 (1.15 to 1.75) respectively, compared to quintile 1. However, this association was attenuated after adjustment for behavioural risk factors (smoking, BMI, physical activity and alcohol)—adjusted ORs (95% CI) (compared to quintile 1) were 1.02 (0.84 to 1.24) for quintile 2; 1.05 (0.86 to 1.29) for quintile 3; 1.20 (0.97 to 1.47) for quintile 4 and 1.17 (0.95 to 1.45) for quintile 5.

**Table 4:** ORs (95% CI) for all-cause mortality according to neighbourhood-level deprivation quintiles in a cohort of British men aged 60–79 years followed up for 12 years

	Number of deaths (%)	Adjusted for age	Further adjusted for individual social class	Further adjusted for smoking, physical activity, alcohol, BMI	Further adjusted for HDL-C, systolic blood pressure and diabetes
Quintile 1 (n=900)	295 (33)	1.00	1.00	1.00	1.00
Quintile 2 (n=918)	318 (34)	1.11 (0.95 to 1.30)	1.1 (0.93 to 1.29)	1.07 (0.91 to 1.25)	1.07 (0.91 to 1.26)
Quintile 3 (n=774)	319 (41)	1.33 (1.13 to 1.55)	1.29 (1.10 to 1.52)	1.20 (1.02 to 1.42)	1.24 (1.05 to 1.46)
Quintile 4 (n=670)	301 (45)	1.48 (1.26 to 1.73)	1.42 (1.20 to 1.68)	1.25 (1.06 to 1.48)	1.29 (1.09 to 1.54)
Quintile 5 (n=662)	312 (47)	1.56 (1.33 to 1.84)	1.49 (1.26 to 1.77)	1.26 (1.06 to 1.50)	1.25 (1.04 to 1.49)
Random effect variance (SE)		0.03 (0.03)	0.03 (0.03)	0.03 (0.03)	0.02 (0.03)
ICC		0.0003	0.0003	0.0003	0.0001
P for trend		<0.0001	<0.0001	0.002	0.002

BMI, body mass index; HDL-C, high-density lipoprotein cholesterol; ICC, intraclass correlation coefficient.

Table 5 presents associations of the separate components of the English IMD with CVD and all-cause mortality. All components, except housing, tended to have a greater risk of mortality at higher deprivation levels, although only some (income, employment and health and disability) were statistically significant associations. CVD mortality risk increased with employment-related deprivation—those in quintiles 4 and 5 had a twofold increased odds of CVD mortality. Income-related deprivation was also associated with an increased CVD mortality risk.

**Table 5:** Age-adjusted ORs ((95% CI) for CVD mortality and all-cause mortality according to components of the English Index of Multiple Deprivation quintiles in a cohort of men aged 60–79 years followed up for 12 years

	Number of deaths (%)	Income	Employment	Health and disability	Education	Housing	Crime	Environment
CVD mortality								
Quintile 1 (n=823)	91 (11)	1.00	1.00	1.00	1.00	1.00	1.00	1.00
Quintile 2 (n=840)	107 (13)	1.01 (0.77 to 1.32)	1.48 (1.04 to 2.12)	1.64 (1.16 to 2.33)	1.03 (0.78 to 1.36)	0.85 (0.68 to 1.06)	1.02 (0.78 to 1.33)	0.91 (0.70 to 1.20)
Quintile 3 (n=666)	113 (17)	1.52 (1.17 to 1.97)	1.73 (1.22 to 2.46)	1.69 (1.20 to 2.37)	1.46 (1.13 to 1.88)	0.82 (0.64 to 1.05)	1.14 (0.88 to 1.49)	1.39 (1.09 to 1.78)
Quintile 4 (n=551)	82 (15)	1.32 (1.00 to 1.75)	2.27 (1.61 to 3.19)	1.95 (1.40 to 2.72)	1.18 (0.89 to 1.56)	0.9 (0.68 to 1.21)	1.32 (1.02 to 1.71)	1.18 (0.89 to 1.55)
Quintile 5 (n=494)	85 (17)	1.54 (1.17 to 2.04)	2.08 (1.46 to 2.96)	1.96 (1.39 to 2.76)	1.33 (1.02 to 1.73)	0.71 (0.51 to 0.99)	1.31 (0.99 to 1.73)	1.32 (0.99 to 1.75)
Random effect variance (SE)		0.03 (0.07)	0.005 (0.07)	0.02 (0.07)	0.02 (0.07)	0.02 (0.07)	0.03 (0.07)	0.02 (0.07)
ICC		0.0003	0.00001	0.0001	0.0001	0.0001	0.0003	0.0001
P for trend		<0.0001	<0.0001	<0.0001	0.02	0.06	0.01	0.01
All-cause mortality								
Quintile 1 (n=823)	275 (33)	1.00	1.00	1.00	1.00	1.00	1.00	1.00
Quintile 2 (n=840)	294 (35)	0.94 (0.80 to 1.11)	1.04 (0.85 to 1.27)	1.13 (0.93 to 1.37)	1.03 (0.87 to 1.22)	0.94 (0.82 to 1.09)	1.03 (0.88 to 1.21)	1.00 (0.85 to 1.18)
Quintile 3 (n=666)	270 (41)	1.28 (1.09 to 1.51)	1.2 (0.99 to 1.46)	1.13 (0.93 to 1.37)	1.17 (0.99 to 1.38)	0.95 (0.81 to 1.10)	1.17 (0.99 to 1.37)	1.3 (1.11 to 1.52)
Quintile 4 (n=551)	240 (44)	1.35 (1.14 to 1.59)	1.48 (1.22 to 1.79)	1.34 (1.11 to 1.61)	1.35 (1.14 to 1.59)	1.04 (0.87 to 1.24)	1.28 (1.09 to 1.50)	1.17 (0.99 to 1.39)
Quintile 5 (n=494)	227 (46)	1.36 (1.14 to 1.61)	1.55 (1.28 to 1.88)	1.48 (1.22 to 1.79)	1.35 (1.15 to 1.59)	0.77 (0.63 to 0.95)	1.2 (1.01 to 1.43)	1.35 (1.13 to 1.61)
Random effect variance (SE)		0.03 (0.03)	0.02 (0.02)	0.03 (0.03)	0.03 (0.03)	0.03 (0.03)	0.04 (0.03)	0.03 (0.03)
ICC		0.0003	0.0001	0.0003	0.0003	0.0003	0.0005	0.0003
P for trend		<0.0001	<0.0001	<0.0001	<0.0001	0.1	0.002	<0.0001

CVD, Cardiovascular disease; ICC, intraclass correlation coefficient.

Table 6 shows associations between individual social class and CVD, and all-cause mortality and the effect of adjustment for neighbourhood IMD. CVD mortality risk increased from higher (social class I) to lower social class groups. However, this increased risk in manual social classes (social classes III manual, IV and V) was attenuated on adjustment for neighbourhood IMD quintiles (table 6).

**Table 6:** Individual social class and cardiovascular disease and all-cause mortality in a cohort of British men aged 60–79 years followed up for 12 years

Social class	Number of deaths (%)	Adjusted for age	Further adjusted for neighbourhood deprivation quintiles
Cardiovascular disease mortality			
I (n=381)	39 (10)	1.00	1.00
II (n=1078)	165 (15)	1.56 (1.11 to 2.20)	1.48 (1.05 to 2.09)
III non-manual (n=400)	52 (13)	1.31 (0.88 to 1.96)	1.17 (0.78 to 1.76)
III manual (n=1600)	246 (15)	1.68 (1.20 to 2.35)	1.42 (1.01 to 2.00)
IV (n=348)	58 (17)	1.77 (1.19 to 2.64)	1.44 (0.96 to 2.16)
V (n=117)	20 (17)	1.94 (1.13 to 3.32)	1.53 (0.88 to 2.65)
Random effect variance (SE)		0.01 (0.06)	0.0009 (0.06)
ICC		0.00003	0.0003
P for trend		0.004	0.24
All-cause mortality			
I (n=381)	126 (33)	1.00	1.00
II (n=1078)	409 (38)	1.16 (0.95 to 1.41)	1.12 (0.92 to 1.37)
III non-manual (n=400)	164 (41)	1.23 (0.98 to 1.55)	1.13 (0.90 to 1.43)
III manual (n=1600)	640 (40)	1.32 (1.09 to 1.59)	1.15 (0.95 to 1.40)
IV (n=348)	157 (45)	1.47 (1.17 to 1.85)	1.25 (0.99 to 1.59)
V (n=117)	49 (42)	1.55 (1.12 to 2.14)	1.26 (0.91 to 1.76)
Random effect variance (SE)		0.04 (0.03)	0.03 (0.03)
ICC		0.0005	0.0003
P for trend		<0.0001	0.08

Since the IMD measures used were not identical in England, Wales and Scotland, the results of sensitivity analyses restricted to 3374 men from England using the English IMD quintiles are presented in online supplementary table S1. These sensitivity analyses showed results similar to those with all men in the cohort. CVD and all-cause mortality risk according to English IMD quintiles was greater in more deprived quintiles. These risks remained statistically significant after adjustment for individual social class and established cardiovascular risk factors (see online supplementary table S1).

## Discussion

In this longitudinal study of older British men aged 60–79 years followed up for 12 years, significant differences in CVD mortality and all-cause mortality risk were observed according to neighbourhood-level socioeconomic deprivation. Older men from more deprived neighbourhoods had a higher CVD mortality risk that was independent of individual (occupational) socioeconomic position. This increased risk in those living in more deprived areas was weakened slightly, but remained, after adjustment for individual socioeconomic position and cardiovascular risk factors including smoking, BMI and physical activity. Of the components of neighbourhood deprivation used, employment, health and disability, and income-related deprivation were associated with an increased risk of CVD mortality.

### Strength and weaknesses

We believe the findings of this paper add considerable evidence to the issue of neighbourhood socioeconomic factors and CVD mortality in older populations; to the best of our knowledge, this is the first report using prospective national data to investigate this issue among older British men. We used longitudinal data with multilevel analyses to take account of the hierarchical nature of the data and adequately estimate the area-level effects; previous studies (summarised in table 1) have not all taken account of the multilevel structure of data, which could lead to narrow confidence limits for estimates and inflated significance levels.^26^ Our results are based on a socially representative cohort of older British men with a high rate of follow-up (nearly 98%). The cohort aged 60–79 years at baseline is a largely stable population; only a very small proportion (<5%) moved from their area of residence during follow-up. We used a combined measure of neighbourhood deprivation (index of multiple deprivation, IMD) for the cohort comprising participants from England, Scotland and Wales, based on a recently proposed method for combining IMD measures for the three countries.^19^ Sensitivity analyses showed similar results for English IMD (the largest proportion of the cohort). However, since our study comprises only white European men, the generalisability of the study to women and other ethnic groups is limited. A cohort of older British women (comparable to our study) observed findings similar to our results, with an influence of neighbourhood socioeconomic factors that was independent of behavioural risk factors.^8^

### Comparison with other studies

Few other studies have investigated prospective associations in older populations between neighbourhood-level socioeconomic deprivation and CVD mortality. Results from the Cardiovascular Health Study with an 8 year follow-up found that the risk of CVD mortality in participants aged >65 years from more disadvantaged neighbourhoods was 1.3 times greater compared with those from less deprived areas after adjustment for individual socioeconomic and cardiovascular factors.^6^ These increased risks in the Cardiovascular Health Study are comparable to our results (1.3 to 1.4 times greater risk of CVD death in older people living in more deprived areas), which was observed after adjustment for individual-level socioeconomic position and cardiovascular risk factors.^6^ Another study in a US population reported a weaker effect of area-level deprivation on CVD mortality in older (55–74 years) compared to younger (25–54 years) age groups.^10^

We also explored the components of neighbourhood deprivation in an attempt to distinguish specific aspects of neighbourhoods that relate to CVD mortality. Employment and income-related deprivation, in particular, were associated with CVD mortality risk in older people. Crime and education-related deprivation were weakly associated with CVD mortality, while housing and living environment were not associated. Employment-related deprivation is likely to be a strong indicator of neighbourhood-level economic deprivation. It is not, however, likely to have a direct effect on this cohort of older men, who were mostly retired at the point of assessment. The weak influence of other components could be due to poor measurement of housing, living environment and crime. The ‘health and disability’ component (based on years of potential life lost, emergency hospital admissions, illness to disability ratio, adults <60 years with mood/anxiety disorders) was associated with an increased mortality risk, although not as strongly as employment-related deprivation. Inclusion of the health domain in the IMD does not appear to affect socioeconomic inequalities in health.^27^

In our study, the increased CVD mortality risk associated with more deprived neighbourhoods was stronger than individual socioeconomic position on adjustment. Greater neighbourhood deprivation was associated with a steady increased risk of CVD mortality, which was evident from quintile 3 onwards; this increased risk was independent of individual socioeconomic position. The increased risk in lower (individual-level) socioeconomic groups, however, was attenuated on adjustment for neighbourhood-level deprivation. Previous analyses in our cohort at an earlier age (52–73 years) using the Carstairs measure of neighbourhood deprivation showed weaker associations with CVD, which were diminished on adjustment for individual socioeconomic position.^28^ The results of the present analyses may reflect a stronger effect of neighbourhood factors in older age. Our results also indicate a stronger association between neighbourhood deprivation and CVD mortality compared to non-CVD mortality—the associations with non-CVD mortality were attenuated on adjustment for behavioural risk factors (smoking, physical activity, BMI).

### Implications and conclusions

The findings of this study highlight the impact of neighbourhood socioeconomic factors on CVD mortality in older populations—older people living in more deprived or disadvantaged neighbourhoods are likely to have a greater risk of CVD mortality. It also highlights that the role of neighbourhood factors in older age is independent of individual socioeconomic position. Furthermore, we observed that neighbourhood deprivation was associated with CVD mortality rather than with CVD incidence (which also included non-fatal CVD cases). These findings suggest that the influence of deprivation may particularly affect survival from CVD and its determinants, implicating the availability and quality of treatment rather than the determinants of CVD incidence (smoking, cholesterol, BMI). Given the small differences in prevalence of diabetes across deprivation groups, diabetes does not appear to account for this association between deprivation and CVD mortality.

Individual-level risk factors including smoking, BMI and physical activity, which are known to be influenced by neighbourhood factors,^29^ made a limited contribution to the increased risk associated with deprived neighbourhoods. The role of other possible contributing factors, including air pollution, diet, cognition and familial factors or social support, merit further research. Additionally, the contribution of specific aspects of neighbourhood deprivation in older people with better measures of housing, living environment, access to healthcare and stress is needed.

**What is already known on this subject**

- Studies, mostly in middle-aged populations, have shown that neighbourhood-level socioeconomic factors are associated with cardiovascular disease (CVD) mortality.
- Evidence from longitudinal studies on the influence of neighbourhood socioeconomic factors in older age on CVD mortality is limited.

**What this study adds**

- In a population-based study of 3924 older British men, CVD mortality risk increased steadily with greater levels of neighbourhood-level deprivation.
- This influence of neighbourhood deprivation was independent of individual-level social class and cardiovascular risk factors.
- Opportunities remain to reduce inequalities in CVD mortality in older age by addressing neighbourhood-level factors.

## Supplementary Material

Web table

## References

[1] Townsend N, Wickramasinghe K, Bhatnagar P, et al Coronary heart disease statistics 2012 edition. London: British Heart Foundation, 2012.

[2] Huisman M, Read S, Towriss CA, et al Socioeconomic inequalities in mortality rates in old age in the world health organization Europe region. Epidemiol Rev 2013;35:84–97. 10.1093/epirev/mxs010 23382476

[3] Borrell LN, Diez Roux AV, Rose K, et al Neighbourhood characteristics and mortality in the Atherosclerosis Risk in Communities Study. Int J Epidemiol 2004;33:398–407. 10.1093/ije/dyh063 15082648

[4] Smith GD, Hart C, Watt G, et al Individual social class, area-based deprivation, cardiovascular disease risk factors, and mortality: the Renfrew and Paisley Study. J Epidemiol Community Health 1998;52:399–405. 10.1136/jech.52.6.399 9764262 PMC1756721

[5] Diez Roux AV, Borrell LN, Haan M, et al Neighbourhood environments and mortality in an elderly cohort: results from the cardiovascular health study. J Epidemiol Community Health 2004;58:917–23. 10.1136/jech.2003.019596 15483307 PMC1732601

[6] Major JM, Doubeni CA, Freedman ND, et al Neighborhood socioeconomic deprivation and mortality: NIH-AARP diet and health study. PLoS ONE 2010;5:e15538 10.1371/journal.pone.0015538 21124858 PMC2990774

[7] Sanchez-Santos MT, Mesa-Frias M, Choi M, et al Area-level deprivation and overall and cause-specific mortality: 12 years’ observation on British women and systematic review of prospective studies. PLoS ONE 2013;8:e72656 10.1371/journal.pone.0072656 24086262 PMC3782490

[8] Steenland K, Henley J, Calle E, et al Individual- and area-level socioeconomic status variables as predictors of mortality in a cohort of 179,383 persons. Am J Epidemiol 2004;159:1047–56. 10.1093/aje/kwh129 15155289

[9] Waitzman NJ, Smith KR Phantom of the area: poverty-area residence and mortality in the United States. Am J Public Health 1998;88:973–6. 10.2105/AJPH.88.6.973 9618634 PMC1508210

[10] Clarke P, Nieuwenhuijsen ER Environments for healthy ageing: a critical review. Maturitas 2009;64:14–9. 10.1016/j.maturitas.2009.07.011 19695800

[11] Ramsay SE, Morris RW, Whincup PH, et al Socioeconomic inequalities in coronary heart disease risk in older age: contribution of established and novel coronary risk factors. J Thromb Haemost 2009;7:1779–86. 10.1111/j.1538-7836.2009.03602.x 20015318 PMC2810435

[12] Chan KS, Roberts E, McCleary R, et al Community characteristics and mortality: the relative strength of association of different community characteristics. Am J Public Health 2014;104:1751–8. 10.1111/j.1749-6632.2009.05333.x 25033152 PMC4151920

[13] Wannamethee SG, Lowe GDO, Whincup PH, et al Physical activity and hemostatic and inflammatory variables in elderly men. Circulation 2002;105:1785–90. 10.1161/hc1502.107117 11956120

[14] Wannamethee SG, Shaper AG, Whincup PH, et al Obesity and risk of incident heart failure in older men with and without pre-existing coronary heart disease: does leptin have a role? J Am Coll Cardiol 2011;58:1870–7. 10.1016/j.jacc.2011.06.057 22018297

[15] Office of the Deputy Prime Minister. The English Indices of Deprivation 2004 (revised). Wetherby: ODPM Publications, 2004.

[16] Scottish Executive. Scottish Index of Multiple Deprivation 2004. Edinburgh: 2004.

[17] Welsh Assembly's Statistical Directorate and the Local Government Data Unit. Welsh Index of Multiple Deprivation 2005. StatsWales, 2005 http://census.ukdataservice.ac.uk/. (Accessed 13/08/2015).

[18] UK Data Service Census Support 2013.

[19] Payne RA, Abel GA UK indices of multiple deprivation—a way to make comparisons across constituent countries easier. Health Stat Q 2012;53(Spring).

[20] Stewart CH Multilevel modelling of event history data: comparing methods appropriate for large datasets. University of Glasgow, 2010.

[21] Yang M, Goldstein H Modelling survival data in MLwiN 1.20. Center for Multilevel Modelling, Bedford Group for Lifecourse and Statistical Studies, Institute of Education, University of London, 2003.

[22] Steele F, Diamond I, Amin S Immunization uptake in rural Bangladesh: a multilevel analysis. J Royal Stat Soc Series A (Stat Soc) 1996;159:289–99. 10.2307/2983175

[23] Goldstein H, Browne W, Rasbash J Partitioning variation in multilevel models. Underst Stat 2002;1:223 10.1207/S15328031US0104_02

[24] Rashbash J, Charlton C, Browne WJ, et al MLwiN Version 2.1. Center for Multilevel Modelling, University of Bristol, 2009.

[25] Leckie G, Charlton C runmlwin: A Program to Run the MLwiN Multilevel Modeling Software from within Stata. J Stat Software 2013;52:1–40.

[26] Goldstein H Multilevel Statistical Models. 4th edn. Wiley. Chichester, 2011.

[27] Adams J, White M Removing the health domain from the Index of Multiple Deprivation 2004—effect on measured inequalities in census measure of health. J Public Health 2006;28:379–83. 10.1093/pubmed/fdl061 17065177

[28] Morris RW, Wannamethee G, Lennon LT, et al Do socioeconomic characteristics of neighbourhood of residence independently influence incidence of coronary heart disease and all-cause mortality in older British men? Eur J Cardiovasc Prev Rehabil 2008;15:19–25. 10.1097/HJR.0b013e3282f11f81 18277181

[29] Diez Roux AV, Mair C Neighborhoods and health. Ann N Y Acad Sci 2010;1186:125–45. 10.1111/j.1749-6632.2009.05333.x 20201871

