# Supplementary material for: The influence of neighbourhood-level socioeconomic deprivation on cardiovascular disease mortality in older age: longitudinal multilevel analyses from a cohort of older British men
Source: J Epidemiol Community Health. 2015 Aug 18;69(12):1224–31. doi: 10.1136/jech-2015-205542 (PMC4680118; doi:10.1136/jech-2015-205542)
Supplement: Web table [file jech-2015-205542-s1.pdf]

Supplementary table – sensitivity analyses restricted to England only

Supplemental table 1: Odds ratios (95%CI) for cardiovascular disease mortality and all-cause mortality according to neighbourhood-level deprivation quintiles – restricted to men from England using English IMD quintiles in a cohort of men aged 60-79 years followed up for 12 years

	Number of deaths (%)	Adjusted for age		Further adjusted for individual social class		Further adjusted for smoking, physical activity, alcohol, BMI		Further adjusted for HDL-C, systolic blood pressure and diabetes	
**Cardiovascular disease mortality**									
Quintile 1 (n=823)	91 (11)	1.00		1.00		1.00		1.00	
Quintile 2 (n=840)	107 (13)	1.34	(1.01,1.79)	1.31	(0.98,1.74)	1.28	(0.96,1.71)	1.21	(0.90,1.63)
Quintile 3 (n=666)	113 (17)	1.73	(1.30,2.29)	1.66	(1.25,2.22)	1.54	(1.15,2.06)	1.56	(1.16,2.10)
Quintile 4 (n=551)	82 (15)	1.56	(1.16,2.09)	1.5	(1.11,2.03)	1.34	(0.99,1.82)	1.31	(0.95,1.80)
Quintile 5 (n=494)	85 (17)	1.83	(1.36,2.45)	1.75	(1.29,2.39)	1.47	(1.07,2.01)	1.38	(1.00,1.91)
Random effect variance (SE)		0.00		0.00		0.00		0.00	
ICC		0		0		0		0	
P for trend		<0.0001		<0.0001		0.02		0.05	
**All-cause mortality**									
	Number of deaths (%)	Adjusted for age		Further adjusted for individual social class		Further adjusted for smoking, physical activity, alcohol, BMI		Further adjusted for HDL-C, systolic blood pressure and diabetes	
Quintile 1 (n=823)	275 (33)	1.00		1.00		1.00		1.00	
Quintile 2 (n=840)	294 (35)	1.16	(0.98,1.38)	1.15	(0.97,1.36)	1.11	(0.93,1.32)	1.07	(0.90,1.28)
Quintile 3 (n=666)	270 (41)	1.26	(1.06,1.50)	1.23	(1.03,1.47)	1.12	(0.94,1.34)	1.11	(0.93,1.33)
Quintile 4 (n=551)	240 (44)	1.47	(1.24,1.75)	1.42	(1.19,1.70)	1.25	(1.04,1.50)	1.27	(1.05,1.53)
Quintile 5 (n=494)	227 (46)	1.56	(1.30,1.86)	1.49	(1.23,1.79)	1.19	(0.98,1.44)	1.17	(0.96,1.42)
Random effect variance (SE)		0.03 (0.03)		0.03 (0.03)		0.03 (0.03)		0.03 (0.03)	
ICC		0.0003		0.0003		0.0003		0.0003	
P for trend		<0.0001		<0.0001		0.03		0.03	
